# The Prevalence of Side Effects of Sinopharm COVID-19 Vaccine: An Experience From Pakistan

**DOI:** 10.7759/cureus.38180

**Published:** 2023-04-26

**Authors:** Taimur Haider, Syeda Rakshan Zehra Abidi, Mehwish Fatima, Aimen Zafar, Rabeeya Qutub Uddin Siddiqui, Wadan Khan, Tazeen Saeed, Adnan Anwar, Atif A Hashmi

**Affiliations:** 1 Pediatrics, District Headquarter Hospital, Jhang, PAK; 2 Forensic Medicine, United Medical and Dental College, Karachi, PAK; 3 Biochemistry, Jinnah Sindh Medical University, Karachi, PAK; 4 Anaesthesiology, Baqai Medical University, Karachi, PAK; 5 Internal Medicine, Bolan Medical Complex Hospital, Quetta, PAK; 6 Internal Medicine, Karachi Medical and Dental College, Karachi, PAK; 7 Physiology, Hamdard College of Medicine and Dentistry, Karachi, PAK; 8 Pathology, Liaquat National Hospital and Medical College, Karachi, PAK

**Keywords:** vaccine side effects, burning at injection site, pain, fever, sinopharm vaccine

## Abstract

Introduction

Vaccination for coronavirus disease 2019 (COVID-19) helps develop protective immunity against COVID-19 without experiencing potentially severe illness. Many vaccines are used worldwide, but there is little data on the efficacy and side effects of the Sinopharm vaccine. Therefore, this study aimed to investigate the reported adverse effects of the Sinopharm vaccine among participants.

Methods

This prospective cross-sectional study was conducted in multiple hospitals in Karachi, Pakistan. The study was eight months, from April 1, 2022, to November 30, 2022. A total of 600 participants who gave informed consent and had received their first and second doses of the Sinopharm vaccine were included in the study. As hypertension and diabetes mellitus (DM) are common prevalent conditions in our population, the duration of DM and hypertension were documented as means and standard deviations apart from age, height, and weight. Side effects of the Sinopharm vaccine were reported as frequencies and percentages.

Results

The study findings showed that out of 600 participants, 376 (62.7%) were males and 224 (37.3%) were females; their mean age was 42.79±14.44 years. Among them, 130 (21.7%) had hypertension, and 138 (23.0%) had DM. All participants received the Sinopharm vaccine. Fever was the most frequently reported adverse effect following the first dose of the Sinopharm vaccine in 308 (51.3% of participants), followed by burning at the injection site in 244 (40.7% of participants) and pain at the injection site in 228 (38.0% of participants). Following the second dose of the Sinopharm vaccine, fever was the most frequently reported side effect in 254 (42.3%) participants, followed by pain at the injection site in 236 (39.5%) participants and burning at the site of injection in 210 (35.0%) participants. Moreover, joint pain in 194 (32.3%), shortness of breath in 170 (28.3%), swelling of glands in 168 (28.0%), chest pain in 164 (27.3%), and muscle pain were reported by 140 (23.3%) participants. The level of satisfaction showed that the majority of the participants, 334 (55.7%), were satisfied, 132 (22.0%) were very satisfied with their vaccination, and only 12 (2.0%) were dissatisfied.

Conclusion

This study concluded that fever was the most frequent side effect after both doses of the Sinopharm vaccine. Pain and burning at the injection site and joint pain were among the other common side effects reported by most participants. The Sinopharm COVID-19 vaccine had mild, predictable, and non-life-threatening side effects after the first and second doses.

## Introduction

The novel coronavirus disease 2019 (COVID-19), also recognized as the severe acute respiratory syndrome coronavirus 2 (SARS-CoV-2) outbreak, has rapidly spread globally. The disease's outbreak has significantly threatened global healthcare systems and financial plans [1]. As of April 16, 2023, more than 763 million confirmed cases of COVID-19 and over 6.9 million deaths were reported globally [2]. The deadly pandemic has spread through most of the world's nations in four waves, and it has produced a variety of clinical indications in the general populace [3]. The highly contagious SARS-CoV-2 has variable mortality outbreak patterns and a high prevalence of transmission [4].

Restriction measures were implemented worldwide to halt the spread of the COVID-19 infection. According to the Iranian Ministry of Health and Medical Education (MoHME), it was announced to restrict traffic based on suspected risk in geographical areas, limit the number of workers at jobs, introduce online working at home, make mask wear compulsory in public areas, and raise public awareness through social media [5]. Every time these limitations were implemented, there was a subsequent drop in suspected cases and fatalities; conversely, ignoring the restrictions and the COVID-19 preventative measures (wearing a mask, keeping oneself isolated from others, and disinfecting one's hands) resulted in a new wave [6]. Despite the numerous pharmaceuticals suggested for COVID-19, more research is still required to establish their efficacy and potency. The best method of disease management may involve developing and administering vaccines [7]. The vaccination is considered an effective long-term solution to eliminating SARS-CoV-2 because it greatly reduces the chance of infection, severity, and fatalities of the disease [8].

Major international biopharmaceutical companies have developed several COVID-19 vaccines. One of the two deactivated viral vaccines in COVID-19 is called Sinopharm, also known as the BBIBP-CorV vaccine. In July 2021, Sinopharm received an emergency use license. At first, the Middle East, Africa, and Asia were the only regions where Sinopharm could be used [9,10]. Sinopharm has 79.3% efficacy after two doses [10]. It may have adverse effects, just like any other medication or vaccine that makes claims of healing and protection.

The Sinopharm vaccine is an inactivated vaccine that delivers SARS-CoV-2 antigens to the body through two injections, separated by 14 or 21 days. The intramuscular injection of an inactivated COVID-19 vaccine uses the virus' dead antigens to produce antibodies that boost the immune system to combat subsequent COVID-19 viral attacks [11]. Traditional whole-virus-inactivated vaccines do not cause symptomatic illness. Using this method, the inactivated viruses in vivo retain their capacity to reproduce while exhibiting minimal or no symptoms [12]. Experimental trials for the Sinopharm COVID-19 vaccine's phases 1 and 2 were conducted in China, with one study for each phase. A COVID-19-counteracting antibody reaction was elicited by the vaccine with a minimal incidence of side effects, according to data from 640 participants. Fever and pain at the injection site were the most reported side effects, although they were minor, self-recovering, and did not require medical attention [13]. With 69 000 participants overall, phase III was conducted over IV trials in developing nations, such as the United Arab Emirates, Egypt, Bahrain, Argentina, Jordan, and Peru. The United Arab Emirates approved the vaccine on December 9, 2020, and the initial findings of its phase 3 trial proposed that the vaccine was 86% effective [9].

Despite the Sinopharm vaccine being widely used in some nations, there is little research on its side effects. Therefore, this study was intended to assess the incidence of reported side effects of the Sinopharm vaccine among participants.

## Materials and methods

This cross-sectional, multi-center study was performed using a non-probability sampling method. Ethical approval was taken before conducting the study. The study was eight months, from April 1, 2022, to November 30, 2022. A total of 600 participants who gave informed consent and had first and second doses of the Sinopharm vaccine administered to them were included in the study. The inclusion criteria were age (above 18 years). Participants with incomplete information, like a history of diabetes mellitus (DM) and hypertension, were excluded from the study. Participants who did not receive a double dose, who had gotten a vaccination with a different vaccine rather than Sinopharm, or who had never received a COVID-19 vaccination were all excluded from the study.

The participant's details were gathered using a pre-designed questionnaire. Demographic information of participants, for instance, gender, age, underlying diseases, Sinopharm vaccine with both doses along with a booster dose, whether or not the participants were infected with COVID-19 infection in the past, and the prevalence of any systemic and local side effects following the first and second doses of post-vaccinations were documented. The level of satisfaction among the participants was also recorded.

The data were analyzed using IBM SPSS Statistics for Windows, Version 26.0. Age, height, weight, and duration of DM and hypertension were documented as means and standard deviations. Frequencies and percentages were reported for demographic factors (sex, vaccine type, number of doses, and local and systemic side effects). As hypertension and DM are common prevalent conditions in our population, the duration of DM and hypertension were documented as means and standard deviations apart from age, height, and weight. Side effects of the Sinopharm vaccine were reported as frequencies and percentages.

## Results

A total of 600 participants received Sinopharm vaccines. Among them, 224 (37.3%) were females, and 376 (62.7%) were males. The mean age of the participants was 42.79±14.44 years. Participants' mean weight and height were 67.43±14.45 kg and 5.51±0.83 ft, respectively. The mean duration of hypertension was 5.24±2.78 years, and the mean duration of diabetes was 5.18±6.618 years. One hundred thirty (21.7%) had hypertension, and 138 (23.0%) had diabetes. At present, 154 (25.7%) participants were infected with COVID-19. Additionally, only 42 (7.0%) participants had previous exposure to COVID-19. Concerning the type of vaccine, all participants received Sinopharm, as shown in Table 1.

**Table 1:** The participants’ basic demographic characteristics (n=600) SD: standard deviation; DM: diabetes mellitus; COVID-19: coronavirus disease 2019

Variables		Values
Age (years), mean±SD		42.79±14.44
Weight (kg), mean±SD		67.43±14.45
Height (ft), mean±SD		5.51±0.83
Duration of hypertension (years), mean±SD		5.24±2.78
Duration of DM (years ), mean±SD		5.18±6.618
Gender, n (%)	Male	376 (62.7%)
	Female	224 (37.3%)
Hypertension, n (%)	Yes	130 (21.7%)
	No	470 (78.3%)
DM, n (%)	Yes	138 (23.0%)
	No	462 (77.0%)
Previous history of COVID-19 infection, n (%)	Yes	154 (25.7%)
	No	446 (74.3%)
Previous COVID-19 exposure, n (%)	Yes	42 (7.0%)
	No	558 (93.0%)

**Figure 1:**
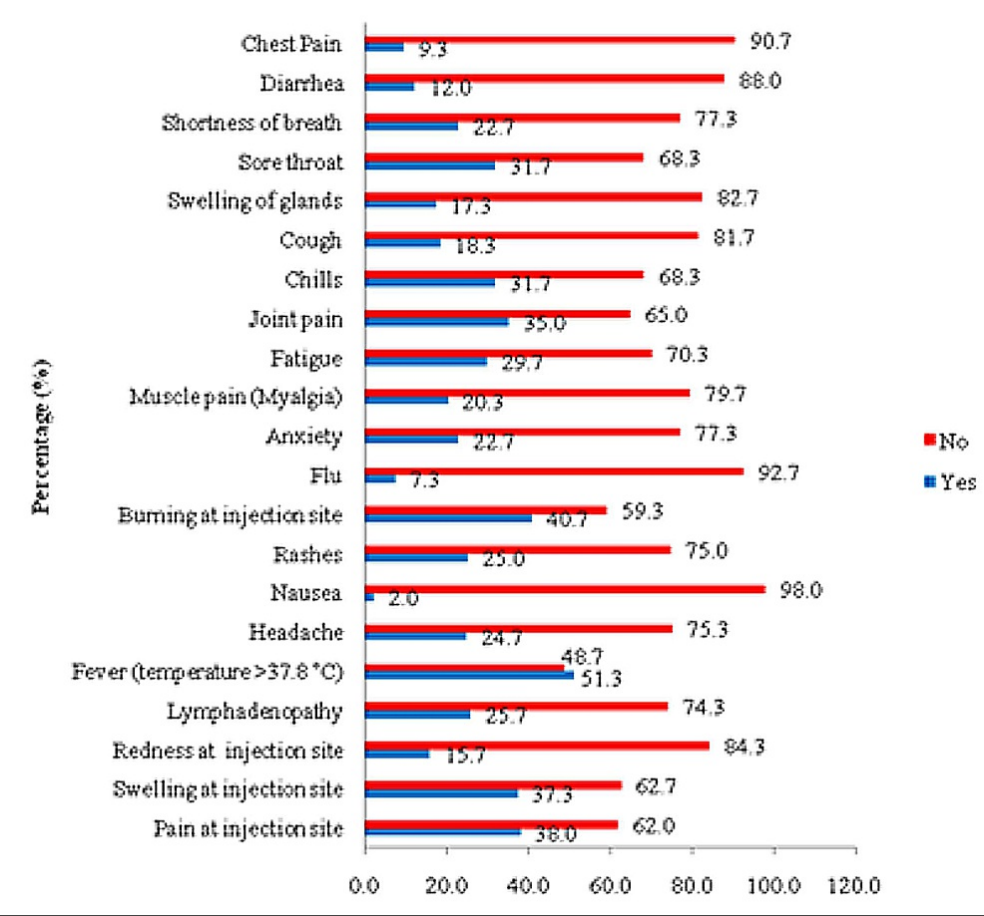
The distribution of adverse effects after receiving the first dose of the Sinopharm vaccine

Fever was the most frequently reported adverse effect following the first dose of the Sinopharm vaccine in 308 (51.3% of participants), followed by burning at the injection site in 244 (40.7% of participants) and pain at the injection site in 228 (38.0% of participants). Moreover, swelling at the injection site was 224 (37.3%), joint pain was 210 (35.0%), chills were 190 (31.7%), and 178 (29.7%) participants reported fatigue. On the other hand, nausea was the least reported side effect by 12 (2.0%) participants, as shown in Figure 1.

**Figure 2:**
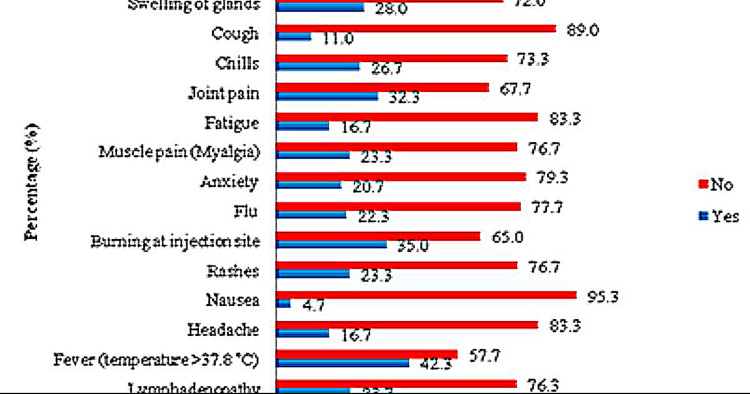
The distribution of adverse effects after receiving the second dose of the Sinopharm vaccine

Following the second dose of the Sinopharm vaccine, fever was the most frequently reported side effect in 254 (42.3%) participants, followed by pain at the injection site in 236 (39.5%) participants and burning at the site of injection in 210 (35.0%) participants. Moreover, joint pain in 194 (32.3%), shortness of breath in 170 (28.3%), swelling of glands in 168 (28.0%), chest pain in 164 (27.3%), and muscle pain were reported by 140 (23.3%) participants. Likewise, nausea was the least reported side effect for the first dose of the vaccine by 28 (4.74%) participants receiving the second dose, as shown in Figure 2.

The level of satisfaction showed that the majority of the participants, 334 (55.7%), were satisfied, and 132 (22.0%) were very satisfied with their vaccination, while only 12 (2.0%) were dissatisfied, as shown in Table 2.

**Table 2:** The level of satisfaction with Sinopharm vaccine.

Variables		Values
		n (%)
Overall subject level of satisfaction with vaccine	Very Satisfied	132 (22.0)
	Satisfied	334 (55.7)
	Don’t know	122 (20.3)
	Dissatisfied	12 (2.0)

## Discussion

The COVID-19 vaccinations have changed the epidemic's trajectory, prevented the loss of tens of millions of lives worldwide, and reduced morbidity and disabilities brought on by COVID-19 infection and its complications [14]. The vaccine protects individuals and communities by limiting the spread of diseases in a community, and vaccines are the safest method currently available for defending people from fatal illnesses [15]. Immunization was crucial in reducing COVID-19 cases and fatalities during the pandemic [16]. Nevertheless, some individuals are concerned about the safety of vaccinations globally. Therefore, the present study illustrated the adverse reactions reported by participants who received the Sinopahrm vaccine.

Meo AS et al., in a study conducted in Pakistan, found that pain at the injection site, overall tiredness, muscle pain, body ache, minimal fever, and headaches were the frequent side effects following doses one and two. After receiving the first dose instead of the second, the subjects reported more severe symptoms and side effects that occurred more frequently [17]. These results were comparable to a study conducted in the United Arab Emirates by Saeed BQ et al., targeted to determine the most frequent Sinopharm side effects. They discovered that following the first dosage of vaccines, 42.2% of participants experienced pain at the injection site, followed by tiredness in 12.2% and headaches in 9.6% of participants. There were no side effects reported by 24.4% of individuals. Moreover, they also found that the most frequent side effects of the second dosage were pain at the injected site (32.6%), tiredness (13.7%), and fatigue (16.3%) [18]. The present study was inconsistent with the previously reported research and showed that participants experienced fever, burning, swelling, and pain at the injection site after receiving both doses.

A study on AstraZeneca, Sinopharm, and Pfizer in Iraq indicated comparable side effects of the vaccine, which revealed reactions at the injection site (54.5%), tiredness (40.9%), fever (37.8%), muscle pain (36.3%), and headache (33.3%) [19]. These findings were not corroborated with the present study, which indicated that the most commonly reported side effects after the first and second doses of the Sinopharm vaccine were fever (first dose: n=308, 51.3%; second dose: n=254, 42.3%). Subsequently, there was pain at the injection site after the first dose (n=228, 38.0%) and the second (n=236, 39.3%).

Likewise, another study by Thonginnetra et al., conducted in Thailand, evaluated the safety of the Sinopharm vaccine among vaccinated recipients. It was observed that pain and tenderness at the site of injection were seen in 37.93% of individuals; fatigue was found in 37.89%; muscular pain was observed in 33.56%; and headaches were reported in 26.76% of individuals. They observed that two dosages of Sinopharm vaccines in adolescents resulted in mild-to-moderate side effects [20]. Similarly, another study in Bahrain investigated the adverse effects of both doses of various vaccines. They noticed that of the four vaccines (Sinopharm, Pfizer-BioNTech, Sputnik, and AstraZeneca), Sinopharm vaccine recipients experienced minimal side effects [21]. The present study did not support the earlier research and revealed that fever, burning, and pain at the injection site were reported side effects. These side effects were minor and required no hospitalization.

Interestingly, the study conducted by Saeed BQ et al., involving 1080 participants, observed that injection site pain and fever were the most common side effects; however, all were temporary and self-resolving, and no treatment was needed [18]. Similar findings were reported in another study [11]. These findings showed similarities with our study and revealed that fever and pain were side effects that were noticed following the two doses of the Sinopharm vaccine.

A study by Riad et al. conducted in Turkey reported that more than 10% of the participants had headaches (18.7%), fatigue (23.6%), and pain at the injection site (41.5%) [22]. The present study was partially similar to the above findings and showed that pain at the injection site was observed after receiving both doses (38.0% vs. 39.3% [first vs. second dose]), whereas fatigue (29.7% vs. 16.7%) and headache were reported in 24.7% after the first dose and in 16.7% after the second dose.

Likewise, another study revealed that the incidence of side effects after receiving the second dose was marginally greater as compared to the first dose, excluding nausea (1.5% vs. 1.1%), allergy (1.1% vs. 0.0%), cough (1.1% vs. 0.7%), intestinal discomfort (1.85% vs. 1.5%), and backache (4.1% vs. 3.0%) [14]. The reaction of the immune system could be used to interpret this result. The immune system may cause symptoms like the flu that persist for a few days after vaccination. Cytokines produced by the immune system may have an inflammatory response involving the vascular system, muscle, and other tissues [23]. These results concurred with those of newly published research [24,25]. These findings were inconsistent with the above-reported studies and indicated that after getting the first dose, adverse symptoms were more common than after receiving the second dose, except for pain at the site of injection (38.0% vs. 39.3% [first vs. second dose]), nausea (2.0% vs. 4.74%), flu (7.3% vs. 22.3%), myalgia (20.3% vs. 23.3%), swelling of the glands (17.3% vs. 28.0%), breathlessness (22.7% vs. 28.3%), diarrhea (12.0% vs. 16.7%), and chest pain (9.3% vs. 27.3%).

Similarly, further research reported that the vaccinated participants had comorbidities such as 7.80% having diabetes and 6.30% having hypertension [18]. The present study was inconsistent with the above-cited studies, indicating that 130 (21.7%) and 138 (23.0%) participants had hypertension and diabetes, respectively.

Similarly, one of the studies performed in Iran studied Sinopharm-vaccinated participants who were older; their mean age was 73.54 years, and 860 (54.9%) participants had co-existing diseases, where the most prevalent disease was diabetes, which existed in 355 cases. Around 979 (62.6%) Sinopharm recipients did not notice any side effects following both doses. Additionally, tiredness, chills or fever, dizziness or headache, and local responses were the most prevalent side effects after receiving the first and second dosages of the Sinopharm vaccine [26]. The present study did not agree with the previously reported study, which reported that the mean age of the participants was 42.79±14.44 years and that comorbidities such as diabetes and hypertension existed in 138 (23.0%) and 130 (21.7%) participants, respectively. Regarding side effects after vaccination, fever was experienced after both doses of the Sinopharm vaccine. Subsequently, there was pain and burning at the site of the injection.

This study had a few limitations as it was a cross-sectional study based on expressed adverse consequences that might be affected by recipients' biases and misconceptions regarding vaccines. Secondly, the sample size was limited compared to the large population that received the vaccine. Moreover, other comorbidities, including DM and hypertension, were not recorded. However, the reality that the Sinopharm vaccine with side effects has received very little research attention to date, despite being the most widely used vaccine in some nations is one of our study's strengths.

## Conclusions

This study concluded that Sinopharm is a safe vaccine for COVID-19 prevention. We found that fever was the most frequently observed side effect after both doses of the Sinopharm vaccines, followed by pain and burning at the injection site and joint pain, among the other common side effects reported by most participants. After both doses, the Sinopharm COVID-19 vaccine showed minor and non-life-threatening adverse effects, and the first dose experienced these adverse effects significantly more frequently than the second dose. Overall, the vaccine was well tolerated, with satisfactory satisfaction among the participants.
